# Neonatal Mortality in Hospitalized Chinese Population: A Meta-Analysis

**DOI:** 10.1155/2019/7919501

**Published:** 2019-01-13

**Authors:** Baoquan Zhang, Yue Dai, Hanqiang Chen, Changyi Yang

**Affiliations:** ^1^Neonatology Department, Fujian Provincial Maternity and Children's Hospital, Affiliated Hospital of Fujian Medical University, Fuzhou 350001, China; ^2^School of Public Health, Fujian Medical University, Fuzhou 350122, China

## Abstract

**Objective:**

In this meta-analysis, we aim to investigate the neonatal mortality in hospitalized Chinese population in the recent 20 years in China, which may provide basis for decreasing the neonatal mortality.

**Methods:**

The merged mortality was estimated based on the random effect model, and subgroup analysis was performed for the gender, publication year, gestational age, and birth weight. Sensitivity analysis was utilized to evaluate the effects of research type and research quality on the effects.

**Results:**

The neonatal mortality in eastern China was lower than that of the central and western China (2.3% versus 2.9; 2.3% versus 26.%). The mortality in neonates with a gestational age of 28-32 weeks (0.6%) was significantly higher than that of <28 weeks (0.1%), 32-37 weeks (0.3%), 37-42 weeks (0.4%), and >42 weeks (0.1%). The mortality in those with a body weight of 1.0-2.5 kg (0.3%) was significantly higher than that of 2.5-4.0 kg (0.2%) and >4.0 kg (0.0%). Sensitivity analysis revealed that the findings of meta-analysis were stable. The major causes for neonatal death included asphyxia, respiratory distress syndrome, and infection.

**Conclusions:**

The hospitalized neonatal mortality showed a tendency of decrease, which differed based on gender, region, gestational age, and birth weight.

## 1. Introduction

Neonatal mortality is an important index for evaluating the perinatal healthcare of a region or even a nation [1]. There are about 3 million neonatal deaths worldwide, which accounts for about 43% in the children aged less than 5 yrs old. The majority is reported from the developing countries [2]. Neonatal period, a time range of 28 days after birth including an early stage (0-7 days) and advanced stage (8-28 days), shows the highest mortality worldwide [3]. Although great stride has been achieved in controlling the childhood mortality aged < 5 yrs, the decrease of neonatal mortality shows a tendency of slow decline. Moreover, in a recent survey, about half of the children aged <5 yrs are neonates [4]. In a survey, the Chinese neonatal death accounts for about 6.4% among the neonatal death worldwide [5]. Nowadays, the total death rate in the neonates is on a decreasing trend with the advances of technical development and social progress; however, in China mainland, neonatal mortality surpassed about 60% of the mortality in children aged < 5yrs [6], among which the mortality was high in the hospitalized cases. It has been reported that neonatal death is associated with the financial status and medical technique [4]. Indeed, there are differences in the medical technique in different regions of China, which triggers variation in the neonatal death. To date, there are some disputes on the neonatal mortality during hospitalization among different studies [7].

In this study, we did this meta-analysis to evaluate the neonatal mortality during the hospitalization in China mainland. Besides, subgroup analysis was performed to investigate the differences in study time, age, region, gestational age, and birth weight.

## 2. Materials and Methods

### 2.1. Strategies

We searched the articles published between 2000 and 2017 by Chinese authors or institutions. The studies published before June 2018 were considered to be eligible for the study. The articles were searched from PubMed, EMBASE database, and three Chinese medical databases including CNKI database, VIP database, and Wanfang databases. The following key words were used for the searching strategy: (death) OR (death rate)] AND (newborn OR neonatal) AND (hospital OR in hospital) AND (China OR Chinese). The articles were manually checked for the information of neonatal mortality. Besides, manual search was performed after reading these articles.

### 2.2. Eligibility

Articles meeting the following criteria were eligible for this study: (i) research studies performed in Chinese neonates involving qualified samples; (ii) studies reporting the mortality of neonates; (iii) reporting the number of live birth and mortality of neonates in a qualified form. The exclusion criteria were as follows: (i) studies based on samples collected from neonates with specific diseases that could not represent the neonatal population; (ii) not reporting complete information; (iii) repeated articles.

### 2.3. Data Collection

The articles were reviewed by two qualified researchers. The data were collected by two independent researchers. In cases of any disputes, a deep communication was performed until consensus. Epidata 3.02 software was utilized for the data entry. For each eligible article, the following information was collected including title, publication year, author information, region, study duration, and number of live births and mortality.

### 2.4. Article Quality Evaluation

The article quality was evaluated using the standards proposed by Agency for Healthcare Research and Quality (QHRQ). The standards used in the cross-sectional study included 11 aspects, including data source, research setting, participants, variables, data, bias, sample size, quantitative variables, statistical analysis, and follow-up [8].

### 2.5. Statistical Analysis

The data were merged using the generic inverse variance model proposed by Sutton et al. Subgroup analysis was performed to analyze the publication year, region, gender, gestational age, birth weight, and neonatal mortality. According to the previous description, we classified the China mainland into three regions (i.e., eastern, central, and western regions). On this basis, we did the sensitivity analysis for the article qualities, to analyze the effects on meta-analysis. The forest plot was drawn using Stata 11.0 software, together with determination of the heterogeneity and publication bias.

## 3. Results

### 3.1. Data Characteristics

**Figure 1:**
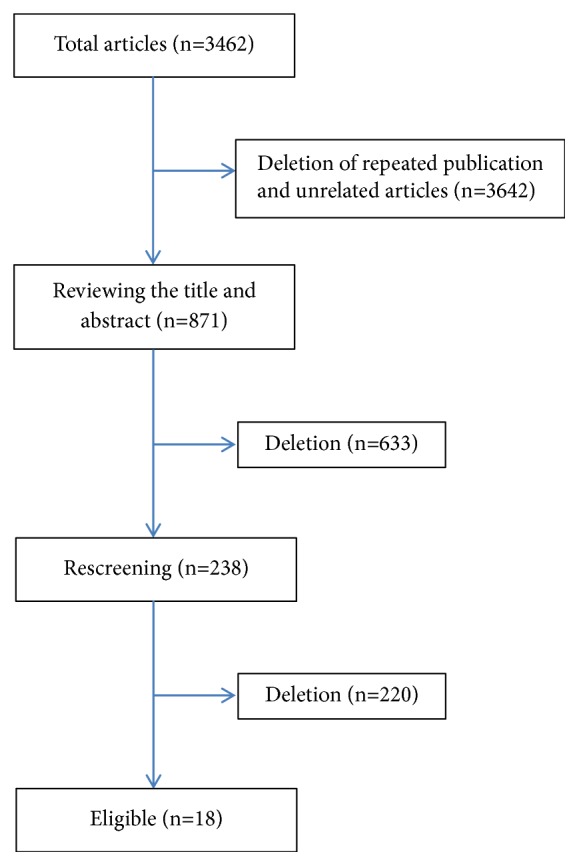
Study flowchart.

A total of 3462 articles were obtained after research. Finally, 18 eligible articles [9–26] involving 488,604 cases were obtained after detailed exclusion (Figure 1). The time range of the studies was between 2000 and 2017. There were 4571 neonatal deaths. The sample volume showed large variances (580-201,115, median: 7030, Table 1).

### 3.2. Methodology Quality Control

A quality score of 4-6 was achieved for the 18 articles. In most studies, the application of variables was clear, and the sample volume was adequate. The major problem for these articles was not mentioning the reasons for data missing and the potential influences. Most of the articles did not report the 95% confidential interval of the neonatal mortality.

### 3.3. Merge of Effect Size

**Figure 2:**
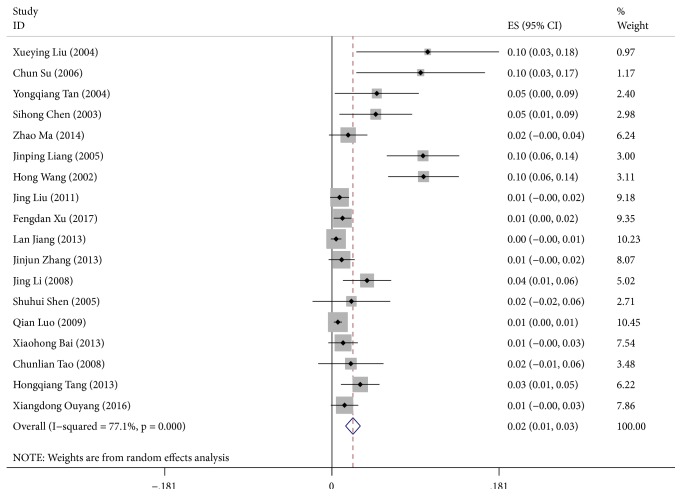
Forest plot for the neonatal mortality in hospital cases.

There was significant heterogeneity in this study (*I*^*2*^=77.10%,* P*<0.001); therefore, the random effect model was used for the merging of effect size. According to the forest plot, the neonatal mortality was 2.3% after mergence (CI: 1.5%-3.1%). Subgroup analysis indicated that the neonatal mortality between 2010 and 2017 was significantly lower than that between 2000 and 2009 (Figure 2). The neonatal mortality of male was higher than that of female. Meanwhile, the neonatal mortality in eastern China was lower than that of the central and western China. The neonatal mortality was the lowest in the full-term newborn infants and those with normal birth weight. The mortality in the neonates with a gestational age of 28-32 weeks and a body weight of 1.0-2.5 kg was higher than the other counterparts, with a ratio of 0.6% and 0.3%, respectively (Table 2).

### 3.4. Sensitivity and Publication Bias

**Figure 3:**
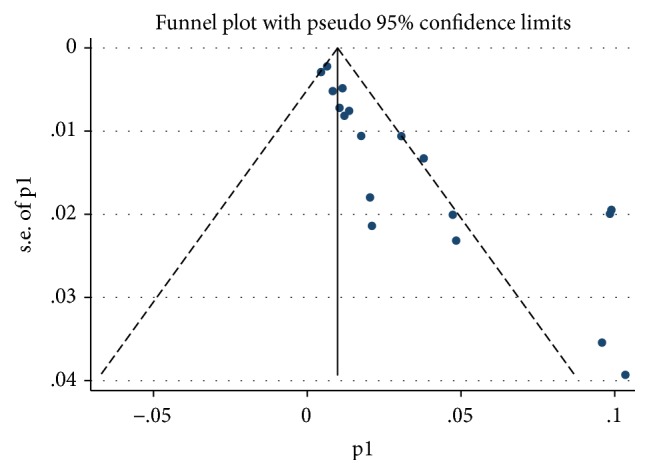
Funnel plot with pseudo 95% CI.

Sensitivity analysis was performed by gradual exclusion of each study. No significant changes were noticed in the merged mortality. These indicated the results for this meta-analysis were stable. Funnel plot revealed the neonatal mortality (Figure 3). The sample size was comparatively low, which resulted in bias to some extent. Begg test indicated a test statistic of 3.48 (P<0.01). Egger test statistic was -0.59 (P>0.01). This implied that there might be publication bias.

## 4. Discussion

Nowadays, more and more attention has been paid to the meta-analysis as it provides high quality evidence. In this meta-analysis, we analyzed the neonatal mortality in Chinese population. Our study contributed to the neonatal researches.

In the recent twenty years, the neonatal mortality decreased from 5.3% (2000-2009) to 1.0% (2010-2017). The merged mortality was 2.3%. According to a recent survey by WHO in 2016, the neonatal mortality dropped from 2.35% in 2008 to 1.92% in 2015, in which the mortality dropped from 0.12% in 2008 to 0.09% in 2015 in Japan, from 0.43% in 2008 to 0.36% in 2015 in US, and from 1.01% in 2008 to 0.55% in 2015 in China [27]. The merged mortality was significantly higher than that reported by WHO. This may be related to the fact that most cases included in this study were neonates in hospital, as well as differences in region, hospital conditions, and sources. In total, the neonatal mortality was higher than that of the developed countries. In this study, the neonatal mortality of male was higher than that of the female, which was in line with the previous study in US [28, 29]. As mentioned in the survey in China mainland in 2010, the neonatal mortality of female was higher than that of the male [30]. As previously described, the risk of death in the neonates born in the regions with high financial income and adequate medical sources was lower, and the mortality may be different in different regions [31, 32], which were in line with our data. The variation in the neonatal death in the eastern and western China is mainly associated with the economic levels in these regions. Our data indicated that neonates with a birth weight of 1.0-2.5 kg and a gestational age of 28-32 weeks showed the highest mortality, which was consistent with the previous description [33, 34]. The prevalence of complications in the neonates was significantly higher than that of the full-term infants, which was mainly associated with low gestational age and birth weight and incomplete organ function that resulted in asphyxia, respiratory distress syndrome, and hemorrhagic disease. On this basis, it is necessary to pay attention to the development of organs, in order to avoid complications. Our data showed that the subgroup mortality in the neonates with a gestational age of <28 weeks and a body weight of <1 kg was the lowest. This may be related to the fact that most of the families gave up the treatment due to financial considerations. The outcome for these cases was death in most cases. This information was not included in the analysis as loss to follow-up.

Among the 18 studies, 17 reported the potential causes for mortality. We selected the top 5 factors from each study; the major causes for mortality were asphyxia, infection/pneumonia, septicaemia, and neonatal respiratory distress syndrome. Similarly, Bale et al. reported these factors were also responsible for neonatal mortality in most developing countries [35]. Besides, in the developing countries, the major causes for neonatal death include asphyxia, preterm labor, and severe infection, accounting for about 70% of the neonatal deaths [35]. In a recent survey aimed to generate high quality data about the burden, timing, and causes of maternal deaths, stillbirths, and neonatal deaths in south Asia and sub-Saharan Africa, the most common causes of neonatal deaths were perinatal asphyxia (40% in south Asia; 34% in sub-Saharan Africa) and severe neonatal infections (35%, in south Asia; 37% in sub-Saharan Africa), followed by complications of preterm birth (19% in south Asia; 24% in sub-Saharan Africa) [36]. In the developed regions, the prime cause for neonatal death is birth defect, followed by preterm labor, sudden death, perinatal complications, and injuries [37–39].

In the developing countries, the lower medical techniques are mainly responsible for the neonatal death. Carlo et al. reported the early neonatal mortality rates decreasing from 11.5 deaths per 1000 live births to 6.8 deaths per 1000 live births after Essential Newborn Care (ENC) training, because of decreases in rates of deaths attributable to birth asphyxia and infection [40]. Late-stage neonatal death in hospital is mainly related to infectious diseases, such as pneumonia and septicemia. Therefore, for the long-term hospitalized neonates, close monitoring should be given to induce the incidence of nosocomial infection such as appropriate application of antibiotics and paying attention to the breast feeding [41].

Sensitivity analysis demonstrated that there might be effects of article quality and research types on the study findings of this study. In future, high quality prospective studies are required to accurately calculate the neonatal mortality. In this study, there is indeed publication bias, and large sample sizes studies are needed to further investigate the neonatal mortality.

There are some limitations for the meta-analysis. The final sample size was not large, and severe mixed factors were available, which may lead to selection bias and information bias. In addition, we only performed subgroup analysis to the gender, gestational age, birth weight, publication year, and region. Neonatal mortality may be related to the treatment options and social or environmental factors, but these factors were not included in the subgroup analysis. We can not exclude the heterogeneity through subgroup analysis. Moreover, there might be differences in the index expression in different articles, which may cause effects on the findings.

In summary, our meta-analysis indicates that the neonatal mortality in hospitalized Chinese population is on a trend of decrease, which demonstrates differences in terms of gender, region, gestational age, and body weight. The major causes responsible for neonatal mortality include asphyxia, RDS, infection, and birth defect. In future, large sample size, multicentered prospective studies are required to obtain the accurate proportion of neonatal mortality.

## Figures and Tables

**Table 1:** Characteristics of eligible studies.

Author	Year	Study duration	Region	Live birth (n)	Death toll (n)	Mortality	Top five reasons for death
Liu et al [9]	2004	1997-2003	Guangdong Province	580	60	0.103	Asphyxia/intracranial hemorrhage/pneumonia/birth defect/RDS
Su et al [10]	2006	1997~2005	Guangdong Province	720	69	0.095	Premature birth/asphyxia/intracranial hemorrhage/pneumorrhagia/RDS
Tan et al [11]	2004	1993~2002	Shanghai	1775	86	0.048	Not available
Chen et al [12]	2003	1995~2002	Guangdong Province	2367	112	0.047	Asphyxia/premature birth/infection/intracranial hemorrhage/scleredema
Ma et al [13]	2014	2003-2012	Guangxi Province	8788	154	0.017	birth defect/septicemia/RDS/asphyxia/bilirubin cerebropathy
Liang et al [14]	2005	1998~2004	Jiangxi Province	2265	223	0.098	Asphyxia/intracranial hemorrhage/RDS/septicemia/scleredema
Wang et al [15]	2002	1995-2000	Sichuang Province	2376	235	0.098	Asphyxia/intracranial hemorrhage/RDS/septicemia/scleredema
Liu et al [16]	2011	2007-2009	Liaoning Province	36861	305	0.008	Premature birth/birth defect/asphyxia/pneumonia/RDS
Xu et al [17]	2017	2008-2014	Beijing	41910	480	0.011	Infection/birth defect/RDS/asphyxia/pneumorrhagia
Jiang et al [18]	2013	1990-2010	Jilin Province	119327	537	0.004	Asphyxia/RDS/birth defect/pneumorrhagia/intracranial hemorrhage
Zhang et al [19]	2013	2003-2012	Jiangsu Province	19158	201	0.010	RDS/birth defect/asphyxia/pneumonia/septicemia
Li et al [20]	2008	2000-2007	Shanghai	5459	207	0.037	Birth defect/RDS/septicemia/pneumonia/asphyxia
Shen et al [21]	2005	1992-2001	Hunan Province	2138	45	0.021	Pneumonia/asphyxia/intracranial hemorrhage/septicemia/tetanus
Luo et al [22]	2009	2005-2008	Chongqing Province	201115	1297	0.006	Asphyxia/premature birth/birth defect/pneumonia/accidental death
Bai et al [23]	2013	2007-2011	Sichuan Province	14909	180	0.012	Pneumonia/asphyxia/birth defect/septicemia/RDS
Tao et al [24]	2008	2003-2007	Guangdong Province	3034	62	0.020	RDS/asphyxia/birth defect/pneumonia/septicemia
Tang et al [25]	2013	2008-2012	Shaanxi Province	8602	263	0.030	Birth defect/RDS/asphyxia/pneumonia/pneumorrhagia
Ouyang et al [26]	2016	2010-2014	Hunan Province	17220	235	0.013	Asphyxia/birth defect/RDS/intracranial hemorrhage/septicemia

**Table 2:** Subgroup analysis for the neonatal mortality.

Variables	Article (n)	Mortality	95% *CI*	*I* ^*2*^	Publication bias	
					Begg's test *p*	Egger's test *p*
Year						
2000~2009	10	0.053	(0.026, 0.079)	86.30%	0.531	0.001
2010~2017	8	0.010	(0.005, 0.014)	12.90%	0.013	0.003
Gender						
Male	18	0.013	(0.008, 0.018)	66.60%	0.000	0.450
Female	18	0.005	(0.002, 0.007)	22.40%	0.000	0.763
Region						
Eastern China	10	0.023	(0.110, 0.034)	56.60%	0.016	0.010
Central China	3	0.029	(0.005, 0.053)	88.90%	0.042	0.063
Western China	5	0.026	(0.004, 0.049)	87.10%	0.042	0.194
Gestational age						
<28 weeks	18	0.001	(-0.005, 0.007)	0.00%	0.085	0.170
28~32 weeks	18	0.006	(-0.000, 0.011)	0.00%	0.020	0.368
32~37 weeks	18	0.003	(0.000, 0.006)	0.00%	0.000	0.577
37~42 weeks	18	0.004	(0.002, 0.007)	0.00%	0.009	0.994
>42 weeks	18	0.001	(-0.004, 0.006)	0.00%	0.074	0.746
Birth weight						
<1000g	18	0.001	(-0.003, 0.005)	0.00%	0.300	0.605
1000~1500g	18	0.003	(-0.001, 0.007)	0.00%	0.002	0.699
1500~2500g	18	0.003	(-0.000, 0.005)	0.00%	0.000	0.263
2500~4000g	18	0.002	(0.001, 0.003)	0.00%	0.005	0.619
>4000g	18	0.000	(-0.003, 0.004)	0.00%	0.005	0.277

## Data Availability

All the data were available upon appropriate request.
